# The safety netting behaviour of first contact clinicians: a qualitative study

**DOI:** 10.1186/1471-2296-14-140

**Published:** 2013-09-25

**Authors:** Caroline HD Jones, Sarah Neill, Monica Lakhanpaul, Damian Roland, Hayley Singlehurst-Mooney, Matthew Thompson

**Affiliations:** 1 Department of Primary Care Health Sciences, University of Oxford, Radcliffe Observatory Quarter, Woodstock Road, OX2 6GG Oxford, England; 2 School of Health, University of Northampton, Park Campus, Boughton Green Road, NN2 7AL Northampton, England; 3 General and Adolescent Paediatrics Unit, UCL Institute of Child Health, 30 Guilford Street, WC1N 1EH London, England; 4 Paediatric Emergency Medicine Leicester Academic Group, Cardiovascular Sciences, University of Leicester, Level G, Jarvis Building RMO, Infirmary Square, LE1 5WW Leicester, England

**Keywords:** Primary health care, Safety netting, Parents, Qualitative research, Acute disease

## Abstract

**Background:**

Acute illness is common in childhood, and it is difficult for healthcare professionals to distinguish seriously ill children from the vast majority with minor or self-limiting illnesses. Safety netting provides parents with advice on when and where to return if their child deteriorates, and it is widely recommended that parents of acutely sick young children should be given safety netting advice. Yet little is known about how and when this is given. We aimed to understand what safety netting advice first contact clinicians give parents of acutely sick young children, how, when, and why.

**Methods:**

This was a qualitative study. Interviews and focus groups were held with doctors and nurses in a general practice surgery, a District General Hospital emergency department, a paediatric emergency department, and an out-of-hours service. Data were analysed using the method of constant comparison.

**Results:**

Sixteen clinicians participated. They described that safety netting advice includes advising parents what to look for, when and where to seek help. How safety netting was delivered and whether it was verbal or written was inconsistent, and no participants described being trained in this area. Safety netting appeared to be rarely documented, and was left to individual preference. Limitations of written materials, and structural barriers to the provision of safety netting, were perceived. Participants described that safety netting was influenced by clinicians’ experience, confidence, time and knowledge; and perceived parental anxiety, experience, and competence. Participants noted several limitations to safety netting including not knowing if it has been understood by parents or been effective; parental difficulty interpreting information and desire for face-to-face reassurance; and potential over-reassurance.

**Conclusion:**

First contact clinicians employ a range of safety netting techniques, with inconsistencies within and between organisations. Structural changes, clinician training, and documentation in patient notes may improve safety netting provision. Research is needed into the optimal components of safety netting advice so that clinicians can consistently deliver the most effective advice for parents.

## Background

Acute illness is one of the most common presentations in children attending primary care, and remains an important cause of child mortality in the UK [1]. In distinguishing the tiny number of children with serious illness from the vast majority with minor or self-limiting illness, clinicians are faced with early, non-specific presentations [2]. Failures or delays in diagnosis cost the NHS millions in legal settlements [1]; and children’s emergency hospital admissions have increased over the past decade, many of which are for minor illnesses which could have been managed in the community [3,4]. Safety netting serves to extend the consultation by providing parents with advice about when and where to return if their child subsequently deteriorates. It could reduce avoidable mortality; and safely reduce re-attendances [5,6].

Safety netting for acutely sick young children is widely recommended by the National Institute for Health and Care Excellence (NICE), Scottish Intercollegiate Guidelines Network (SIGN) and other national groups [6-10], as well as general practitioners (GPs) [11] and researchers [12]. Yet very little is known about what safety netting advice clinicians give, in what format, and when [13]. Addressing this clear knowledge gap will begin to fulfil a vital missing component in the management of acutely sick young children.

This study is part of the Acutely Sick Kids – Safety Netting Interventions for Families (ASK SNIFF) project. The ASK SNIFF project aims to co-develop effective safety netting interventions for parents of acutely sick young children which could be delivered by health professionals. In order to design effective interventions it is important to know what current practice is, and what factors influence clinicians’ provision or choice of safety netting advice. In this study, we aimed to understand the safety netting behaviour of first contact clinicians, including what safety netting advice they give, how, when and why. Due to the lack of prior knowledge on this topic, we used a qualitative study design, which enables exploration of the topic from the perspectives of participants and what they perceive to be important, without being constrained by preconceived hypotheses. Such approaches generate comprehensive and in-depth understanding.

## Method

Our method was influenced by grounded theory (GT) methods, adopting several of the key components of GT. Purposive maximum variation sampling was used, in order to include both doctors and nurses across a range of different first contact care settings and capture the range of experiences of clinicians seeing children in different settings. Recruitment continued until we had included participants from general practice, emergency departments (EDs) and the out-of-hours service (OOHS). At each site we conducted focus groups and/or interviews, according to availability of participants, so that we could include data from focus groups (where participants discuss issues amongst each other leading to elaboration and greater understanding), as well as individual interviews (where participants do not have to share details with colleagues and may speak more freely).

Recruitment was coordinated by email using the local Primary Care Research Network and Comprehensive Local Research Network. Within each participating setting, information about the study was distributed to all doctors and nurses, and those who volunteered to participate gave written informed consent. Two experienced female researchers (a children’s nurse lecturer and a social scientist) conducted focus groups (together) and interviews (separately). They lasted around an hour and were held at clinicians’ workplaces or homes between June-December 2012. They began with an opening question followed by prompts, shown in Table 1. One researcher led the focus groups and the other took notes and gave a closing verbal summary, asking participants to correct any misinterpretations. Focus groups and interviews were audio recorded, transcribed verbatim then anonymised.

**Table 1:** Topic guide for focus groups and interviews

Opening question	What safety netting information do you give parents/carers who seek help for an acutely sick child under 5 years of age?
Prompts	How often do you give safety netting information?
	What, if anything, affects whether or not you give safety netting information?
	(If you give safety netting information) how do you give it?
	For what problems do you most often provide safety netting information for parents?
	What factors influence your choice of safety netting advice?
	How helpful do you feel this information is to parents?

Data were analysed using the GT method of constant comparison, facilitated by QSR NVivo 10. Line by line analysis of transcripts, whilst listening to the audio recordings, generated descriptive (substantive) codes (nodes in NVivo), to which segments of transcripts were assigned. Codes were grouped together as relationships between codes were identified and explored. As analysis progressed, codes were checked for ‘fit’ (in GT terms) with the data and edited/combined or ‘refit’ as necessary, new codes were added, relationships between codes were further explored and subthemes and themes were developed. The coding system was developed by a non-clinical researcher and a children’s nurse lecturer, and agreed amongst the wider research team. Text was allocated to codes by one researcher and checked by another. Emerging themes were discussed amongst all authors.

Approval to conduct the study was obtained from the East Midlands – Nottingham 2 NHS Research Ethics Committee (REC reference 12/EM/0076), and the appropriate research and development offices of each local Trust.

## Results

### Participants

Focus groups and/or interviews were held in one general practice surgery, a District General Hospital (DGH) ED (serving adult and paediatric patients), a paediatric ED, and an OOHS (see Table 2). Ten doctors and six nurses participated, including males and females, of white and south Asian ethnicity, all aged 30 years or older (see Table 3). Four main themes emerged during data analysis: 1) what safety netting clinicians give and how; 2) quality of safety netting, 3) when clinicians give safety netting and why, 4) limitations to safety netting. These are discussed in turn below. Additional file 1 summarises the coding scheme and the subthemes (tree nodes) and codes (nodes) within each theme.

**Table 2:** Summary of participants who took part in focus groups and interviews in each setting

**Setting**	**General practice**	**District general hospital emergency department (adult and paediatric patients)**	**Paediatric emergency department**	**Out of hours service**
**Composition of focus group**	3 practice nurses and 2 GPs	2 nurses and 2 doctors	4 doctors	
**Participants interviewed**		1 doctor		1 GP;1 nurse practitioner
**Total number of participants**	5	5	4	2

**Table 3:** Characteristics of participants

**Characteristic**	**Number of participants**
Male	10
Female	6
White British	13
South Asian	3
Aged 30–39 years	7
Aged 40–49 years	7
Aged ≥50 years	2

### What safety netting clinicians give and how

Participants described their safety netting advice containing information on serious symptoms to look for, where to go for help, and expected illness trajectory; as well as general reassurance that parents can contact a healthcare professional at any time if they are worried. Some showed parents symptoms and signs to look for in pictures, video, or on the child themselves. They signposted parents back to themselves, to other services (general practice, OOHS, ED, pharmacists, NHS direct), and the patient.co.uk website. Other safety netting techniques were referral to community nurses, planning a review and booking an open appointment.

There was no consensus between participants regarding format; the benefits of both written and verbal information was mentioned: *“you give the written information out and that’s brilliant because you don’t take it all in when someone’s telling you”* (DGH ED nurse); *“you don’t actually know how much of that leaflet they’re gonna actually understand, take in, comprehend… going through things step by step, listening, understanding and explaining, I think is more beneficial”* (paediatric ED doctor).

There was no standardisation in the provision of either written or verbal safety netting, within or between organisations: *“we’ve probably all got our favourite patient information leaflets that we give… not at the moment standardised across the practice”* (GP surgery doctor); *“we don’t formalise that in any way it’s just verbal, sort of person to person, you know. I don’t know what you say, or you say, or you say. I know what I say but you know, do we all say the same thing?”* (paediatric ED doctor).

Participants lacked knowledge regarding whether/what safety netting advice is given by their colleagues. Only one described documenting safety netting in patient notes, and acknowledged that: *“the only safety netting I’ve documented is “SOS if symptoms worsen, see GP if no better”… I haven’t got any record of what I actually verbally said apart from that… even that little shorthand is open to interpretation”* (OOHS nurse). No participants described being trained in safety netting, and one specifically mentioned a lack of training: *“certainly I’ve never had anything from the… organisation, any guidance on that whatsoever”* (OOHS doctor).

### Quality of safety netting

Parent information leaflets available through patient.co.uk were commonly described and perceived to be comprehensive, specific, and written in parent-friendly language. Yet clinicians identified that leaflets are not universally appropriate. They were available in multiple languages, but not perceived to be suitable for those who are illiterate or whose first language is not English. Leaflets were also not tailored to different age groups, for example the head injury advice leaflet was not relevant to young children: *“a young child is not going to complain of dizziness… a parent… will be reading it and thinking well, you know, what has this got to do with my child?”* (paediatric ED doctor).

Participants perceived there to be various structural barriers to safety netting services. Open appointments were prevented or discouraged, and arranging follow-up with community nurses was time-consuming. Good computer systems enabled quick access to printable information leaflets; but this was hindered by printers not always working. Lack of consistency between services was perceived to be a barrier to high-quality safety netting: *“the problem is then they go to their GP and they get a slightly different looking leaflet and then come to us and get something slightly different and then they are unsure what that person has said”* (paediatric ED doctor). Inefficient continuity between in-hours services and OOHSs was mentioned by staff in both settings, in relation to clinicians and parents having difficulty accessing re-consultations.

### When clinicians give safety netting advice and why

Participants described the content and detail of safety netting advice varying according to the nature of the illness. Additionally, an array of clinician- and parent-related factors was influential. For example, clinician experience and confidence were perceived to impact the safety netting advice given: *“I think it’s very difficult, sort of different people, different stages of training and also different stages of experience as well and therefore to give that consistent message”* (paediatric ED doctor). Greater clinician experience was thought to increase the attention given to safety netting because: “*the more things you’ve seen go wrong*” (OOHS doctor). Clinicians commonly felt that becoming parents themselves improved their safety netting: “*I would imagine it would increase the amount of safety netting. I think you just, when you’ve got your own children, you’ve been through that, you’ve seen the other side, you know, you can understand the position can’t you, empathise so much more… it has made me a much better GP, much safer, much more considerate, empathic, by being a parent*” (OOHS doctor).

General practice clinicians were frank about the potential shortfall in safety netting when time is limited. Safety netting was thought to vary between in-hours and OOHSs, partly due to greater time availability in OOHS, but also due to the importance attributed to safety netting: *“you feel as though you’re seeing a poorlier group of patients… I would imagine… that the quality of safety netting in general would be better from the doctors that work out of hours shifts”* (OOHS doctor).

Clinicians’ lack of knowledge of a particular condition impeded safety netting, as did lack of awareness of available materials. Some participants, when asked, were not aware of the NICE fever guidance leaflets for parents; others were aware, but no clinicians said they used them. Factors other than awareness and availability of leaflets influenced their use: paediatric ED participants used the head-injury advice leaflet more consistently than other available leaflets partly because: “*it’s probably the longest standing leaflet, is that people have been giving out head injury leaflets since time began”* (paediatric ED doctor). At the DGH ED, the lack of a paediatric emergency consultant was suggested to be responsible for clinicians being: *“a little behind the times in the actual handing out written information… I think probably it’s a case of taking your eye off the ball… not had people available to concentrating, focusing on that area”* (DGH ED doctor).

Perceived parental anxiety and experience influenced safety netting: *“if it’s a young mother and it’s their first child I’ll probably spell things out a bit more and be more specific”* (OOHS nurse). In addition to younger and first-time parents, those of ethnic minority backgrounds who may have difficulties understanding English were given a *“more sort of rigorous approach”* (OOHS doctor). Clinicians judged parental competence informally. Children of those judged to be unable to understand and follow safety netting advice were admitted to hospital. It was commented that these judgements were not always correct, and that knowledge should not be assumed, especially in medically-trained parents.

One DGH ED doctor contradicted the perception that safety netting is inconsistent, and influenced by clinician- and parent-related factors. He described giving safety netting to every patient, and when asked what factors influence his choice of advice he answered: “*no it’s standard, services available are GPs or us*”. This ‘standardisation’ refers to the services parents are signposted to, but not any other information they are given; and he agreed that the format of safety netting (written or verbal) was not standardised.

### Limitations to safety netting

Participants highlighted that they do not know how effective safety netting is: *“we don’t know how much we empower parents to look after their children… often we do seem to see them time and time again”* (GP surgery nurse). They also do not know how well parents understand the advice: *“It’s very difficult to know ‘cause often they’ll nod their heads and say “yes I understand everything you say” and walk off and they might have no idea what we’ve just said”* (DGH ED nurse). Clinicians were reassured when parents demonstrated understanding during consultations, for example repeating information.

The broad and “*grey*” nature (paediatric ED doctor) of childhood illness, and the inability to educate parents on all conditions, were perceived to be problematic. Furthermore, parents may not be able to interpret certain signs and symptoms: *“Drowsiness is quite a common one, the younger the children are, though, the more difficult that is to get across because it’s normal for babies to sleep for quite long periods”* (OOHS doctor).

A major problem perceived by participants is that safety netting is not able to fulfil what they believe to be a primary reason for parental consultation – face-to-face reassurance from a professional: *“it’s permanent paranoia isn’t it… I don’t think you can safety net against that ‘cause no one can tell you 100% that your children aren’t going to die in their sleep”* (DGH ED nurse). Indeed, clinicians reported that they would not want to discourage worried parents from bringing in their children, and one hinted at the risk of leading parents to delay re-consultation: “*I’ve had a few cases recently where I’ve been quite surprised that the parents have left it so long… I wonder if the parents are over-reassured”* (paediatric ED doctor).

## Discussion

There is wide agreement that parents of acutely sick children should be given carefully worded safety netting advice [6-12]; yet the content, format, and delivery of this advice have not previously been documented. This is the first study to our knowledge to explore the safety netting techniques that front line clinicians use when caring for acutely sick young children.

We found consensus among participants that safety netting contains information on what to look for, where and when to seek help; but there was a notable lack of consensus regarding the optimal format and how it should be delivered. Clinicians were unaware of their colleagues’ safety netting behaviour, documentation was variable and no training was described. Safety netting was left to the preference of individual clinicians, and was influenced by an array of parent- and clinician-related factors. Notably, particular attention was given to safety netting for first-time parents, young parents, and those who may have difficulty understanding English (particularly ethnic minority parents). Clinicians described admitting children to hospital when they perceived their parents to be unable to understand and follow safety netting advice; perhaps this contributes to the continuing rise in emergency hospital admissions for minor illnesses [3,4]. It should be noted that one participant contradicted these themes and described safety netting as being standard, rather than inconsistent, at least in terms of signposting parents to services.

Using a modified Delphi approach to seek clinical consensus among GPs and paediatricians, Almond and colleagues [14] recommended five core features of safety netting, namely communicating the existence of diagnostic uncertainty; what to look for; how to seek help; expected time course; and documentation in medical notes. Three of these were described in this study (what to look for, how to seek help, expected time course); but no participants described communicating uncertainty, and just one reported documenting safety netting in patient notes, which he admitted was brief. Similarly to our study, Almond et al. [14] failed to find consensus on whether safety netting should be written or verbal.

Lack of consensus on the format and delivery of safety netting may result from clinicians tailoring advice according to different settings, parents and conditions. It may also be due to clinicians not being training in this area, despite the importance attributed to safety netting by numerous groups, and the fact that it was first described over 20 years ago [15]. Participants in this study felt that experience led them to attribute greater importance to safety netting, suggesting that lack of training may be particularly problematic for junior doctors.

One previous study examined the frequency of safety netting, in 220 parents of feverish children who made 570 contacts with urgent care services. Eighty-one per cent of parents recalled being given safety netting advice. According to patient notes of those who were advised to stay at home, 57% were given advice about what to look for, and 74% were advised who to contact [6]. Our study builds on this by describing the nature of safety netting advice, and the rationale for its use/non-use.

We currently do not know how effective safety netting is or what the effective components are [13,16]. Research is now needed into the effectiveness of different safety netting techniques on outcomes such as referrals, admission rates, re-attendance to health care, as well as parental understanding, anxiety and satisfaction.

### Strengths and limitations

The extent to which the behaviour reported by participants corresponds with what they do in practice is unknown. We do not think that participants generally reported socially desirable responses, because they were explicit about inconsistencies and limitations in their safety netting behaviour; and because similar themes arose in individual interviews (where participants have greater anonymity) compared to focus groups. Having focus groups composed of clinicians from the same organisation may have prevented some from speaking openly. However, we feel that this approach facilitated detailed discussion, leading to a deeper and richer understanding; and we supplemented the data with individual interviews.

Maximum variation sampling ensured participants were included from a range of first contact services, which limited bias; but the extent to which these findings are generalizable to other groups and locations is not known. Participants may have a stronger commitment to or interest in safety netting compared to their peers, or be more critical of safety netting techniques, having agreed to participate. We did not observe notable differences in opinions between genders, ethnic groups, or doctors/nurses, but this should be explored further in larger samples. There were no participants under the age of 30 years, who may have had different perspectives, particularly on training.

Data were collected and analysed by one non-clinical researcher and one healthcare professional: their different perspectives and assumptions led to a richer understanding. The non-clinical researcher was able to examine the data without pre-conceived ideas and assumptions about safety netting, whilst the healthcare professional was able to interpret the participants’ statements in context.

### Implications for research and practice

#### *Structural improvements*

The results presented here suggest structural/organisation changes which could be achieved relatively easily to enhance safety netting. These include improving clinicians’ ability to book open appointments and arrange community nurse follow-up; better continuity and information-sharing between in-hours services and OOHSs; and consistent access to efficient computer systems and working printers. Lack of continuity in OOHS has been highlighted previously [17] and a recent review of the UK OOHS recommended integration with other systems (for example EDs) to ensure integrated care pathways [18]. Additionally, appointing somebody responsible for safety netting may prevent it being neglected, and publicising available safety netting materials may lead to wider use.

#### *Improvements to format and delivery*

Improvements could be made to written materials, for example tailoring leaflets to different age ranges. Better provision is needed for those with low literacy or who are unable to understand written English: a recent study in emergency care reported that 14% of families could not read English [19], which was highlighted as a concern in this study. Previous studies have demonstrated greater knowledge and satisfaction in parents given verbal and written health information, compared to verbal only, when their children were discharged from hospital [20]. Yet written information alone may not be adequate: a systematic review of interventions to influence consulting and antibiotic use with children found that including cartoons or illustrations is more effective than text only [21]. Perhaps there is a need for other mechanisms of delivery for safety netting information in addition to verbal and written leaflet form. Alternative ways of delivering information to parents in an accessible format should be considered, for example use of visual/audiovisual techniques.

#### *Greater consistency*

Lack of consistency and standardisation within and between services was strongly emphasised by participants and thought to be problematic, which matches parents’ desire for explicit and consistent information [5]. However, as highlighted above, safety netting may be more effective when tailored to the individual setting, condition and parent. Furthermore, evidence is lacking on optimal format and delivery mechanisms. Yet there is a need for some consistency, at least in how safety netting is recorded in patient notes; not least because this would enable effective auditing of safety netting provision as recommended by NICE [8]. As discussed above, a previous study found that 57% of feverish children’s medical records had a record of providing parents with information on what to look for, and 74% had a record of providing parents with information on who to contact [6]. Our results suggest that documentation of safety netting is sparse and non-standardised, and that auditing patient records cannot accurately determine how often safety netting actually occurs (without being documented). Standards should be set for documentation of safety netting provision in patient notes.

#### *Clinician training*

Consistent training in safety netting may be beneficial, particularly for junior doctors/nurses who are perhaps more likely to lack experience [22], parental status and confidence, all of which were recognised to impact safety netting. Furthermore, training may improve documentation of safety netting [23]. Training should include warning clinicians about the dangers of falsely assuming parental knowledge, and of over-reassuring parents, both of which were highlighted by participants in this study. For example, only half of children with meningococcal disease were sent to hospital after their first primary care consultation; over-reassurance by clinicians, or false reassurance due to the absence of “red flag” symptoms, may prevent timely re-consultation [2].

## Conclusions

First contact clinicians treating acutely sick young children employ a range of safety netting techniques. There are inconsistencies in safety netting provision, with delivery being left to individual clinician preference and being influenced by a range of parent- and clinician-related factors. Structural and organisational changes, improvements in format and delivery, training, and documentation in patient notes may improve safety netting provision.

## Competing interests

The authors declare that they have no competing interests.

## Authors’ contributions

The study was conceived and designed by SN, ML, DR and MT. SN and HS collected the data. CJ and SN analysed the data. The paper was drafted by CJ; all authors read and approved the final manuscript.

## Pre-publication history

The pre-publication history for this paper can be accessed here:

http://www.biomedcentral.com/1471-2296/14/140/prepub

## Supplementary Material

Additional file 1 Summary of the coding scheme. Click here for file
