# Supplementary material for: The safety netting behaviour of first contact clinicians: a qualitative study
Source: BMC Fam Pract. 2013 Sep 25;14:140. doi: 10.1186/1471-2296-14-140 (PMC3849506; doi:10.1186/1471-2296-14-140)
Supplement: Additional file 1 — Summary of the coding scheme. [file 1471-2296-14-140-S1.doc]

Additional File 1: Summary of the coding scheme

| **Themes** | **Subthemes** | **Codes / Nodes** |
| --- | --- | --- |
| What safety netting is given and how | Content  Actions  Format | Expected illness trajectory  Symptoms to look for  Show signs and symptoms  Signposting  Reassurance  Book an open appointment  Book a review  Community nurses  Written  Verbal  Documentation  Training |
| Quality of safety netting | Consistency  Resources | Consistency/standardisation  Continuity between services  Language  Information not relevant  Speed of access to resources  Printing facilities |
| Factors influencing the safety netting clinicians give (when do clinicians give safety netting advice and why) | Child-related factors  Parent-related factors | Child age  Nature of illness  Educational level  Confidence/experience  Ethnicity  Language  Age  Clinician status  Number of children  Anxiety |
|  |  |  |
|  | Clinician-related factors | Gut feeling, worry  Experience/confidence  Parental status  Knowledge of condition/illness  Knowledge of available materials  Time available  Type of service (in hours, out of hours, emergency)  Staff available to focus on safety netting |
| Limitations to safety netting | Nature of childhood illness  Lack of knowledge on effectiveness  Parental needs | Range of illnesses  Grey nature  Interpretation of advice  Do not know if parents have understood  Do not know if safety netting works  Reassurance |
